# Adiponectin: an adipocyte-derived hormone, and its gene encoding in children with chronic kidney disease

**DOI:** 10.1186/1756-0500-5-174

**Published:** 2012-04-03

**Authors:** Manal F Elshamaa, Samar M Sabry, Marwa M El-Sonbaty, Eman A Elghoroury, Nahed Emara, Mona Raafat, Dina Kandil, Gamila Elsaaid

**Affiliations:** 1 Pediatric Department, National Research Centre, 33 El-Behous street, Dokki,PC:12311, Cairo, Egypt; 2 Pediatric Department, Faculty of Medicine, Cairo University, Cairo, Egypt; 3 Child Health Department, National Research Centre, Cairo, Egypt; 4 Clinical & Chemical Pathology Department, National Research Centre, Cairo, Egypt; 5 Medical Biochemistry Department, National Research Centre, Cairo, Egypt

**Keywords:** Adiponectin, Single nucleotide polymorphisms, CKD, Children, Inflammation

## Abstract

**Background:**

The prevalence of cardiovascular disease (CVD) and inflammation is high in patients with chronic kidney disease (CKD). Adiponectin (ADPN) is an adipocytokine that may have significant anti-inflammatory and anti-atherosclerotic effects. Low adiponectin levels have previously been found in patients with high risk for CVD.

**Methods:**

On seventy eight advanced CKD (stages 4 and 5) pediatric patients undergoing maintenance hemodialysis( MHD) or conservative treatment (CT) the following parameters were studied: body mass index, left ventricular mass index(LVMI), serum adiponectin , cholesterol, HDL-cholesterol, high sensitivity C-reactive protein (hs CRP),interleukin 6(IL6) and single-nucleotide polymorphisms (SNPs) in the ADIPOQ gene at positions 45, and 276. Seventy age-and gender-matched healthy subjects served as control subjects**.**

**Results:**

Markedly (*P =* 0.01) elevated plasma adiponectin levels were observed in CKD patients, especially CT patients, compared to control subjects. The wild type of ADIPOQ 45T > G (T) allele is the main gene for patients and controls. MHD and CT patients had significantly higher frequency of the TT genotypes of +276G > T gene (*P* = 0.04) compared with control subjects. A significant positive correlation was observed between plasma adiponectin and IL6 level, whereas negative correlations were found between adiponectin level, cholesterol, HDL cholesterol and hs CRP. In a stepwise backward multiple regression model only IL6 (*P* = 0.001) was independently associated with plasma adiponectin levels. The adiponectin gene the 276 GT+TT genotypes were associated with a higher level of adiponectin .

**Conclusions:**

The present study demonstrated that ADPN is related to several metabolic and inflammatory CV risk factors in a manner consistent with the hypothesis that this protein might have a protective role against these factors. We observed an association between the +276G>T SNP in the adiponectin gene and CKD in children. Genetic variation of +276 gene seemed to have a positive impact on circulating adiponectin levels in CKD patients.

## Background

Endothelial dysfunction is one of the most serious complications that occur in children with chronic kidney disease (CKD). It is caused by nontraditional risk factors such as inflammation and oxidative stress [1]. Adiponectin (ADPN) seems to play a protective role in vascular injury, as it modulates endothelial inflammatory response in vitro [2] and inhibits endothelial nuclear factor kappa B (NF-kB) signaling [3].

CKD is a unique condition in that exceedingly high incidence of enhanced cardiovascular disease (CVD) events [4-6] [increasing from conservative (CT) to maintenance hemodialysis (MHD) treatment [7]] is paradoxically associated with elevated plasma adiponectin. Although Zoccali et al. [7] have questioned the previously reported protective cardiovascular impact of adiponectin in earlier stages of renal disease [5], available evidence indicates that highest total adiponectin increments are associated with lower cardiovascular risk in end-stage CKD [7-9]. The above observations suggest that adiponectin-dependent metabolic and cardiovascular protection is preserved in advanced chronic uremia, although its effectiveness could be blunted by additional CKD-associated alterations and risk factors. CKD-associated hyperadiponectinemia could be due at least in part to reduced glomerular filtration rate and passive accumulation [10].

Adiponectin is a 30-kD adipocyte complement-related cytokine encoded by the adipose most abundant gene transcript 1 (apM1), which is located in a diabetes susceptibility locus on chromosome 3q27 [11]. Adiponectin acts peripherally and circulates in human plasma at high levels (about 0.01% of the total plasma protein pool) [12]. This protein is thought to have significant anti-inflammatory, insulin-sensitizing and anti-atherogenic effects [13,14].

Adiponectin levels have a strong genetic component, with an additive genetic heritability of 46% [15]. The adiponectin gene (ADIPOQ) consists of three exons and two introns spanning a 17-kb region [16]. The ADIPOQ was found to be the only major gene responsible for plasma adiponectin [17]. Two single nucleotide polymorphisms (SNPs) in ADIPOQ gene were found to be associated with type 2 diabetes in Japanese individuals [18]. One of the SNPs (TG in codon 276) was also associated with lower plasma adiponectin levels, although this relationship was limited to obese subjects [19]. Another SNP (T>G at nucleotide 94: SNP 45) was found to be associated with insulin resistance, and dyslipidemia in a German population [20].A haplotype defined by these two SNPs has also been associated with components of the insulin resistance syndrome in Caucasians [20].

Low plasma levels of adiponectin have been documented in various non renal patient populations at risk of CVD, such as in patients with, essential hypertension [21,22], and coronary artery disease [23,24]. No data on the impact of genetic variations are yet available in pediatric end stage renal disease (ESRD) patients.

The objectives of the present study were, therefore, to relate plasma levels of adiponectin in pediatric CKD patients to body mass index (BMI), inflammatory biomarkers and estimated glomerular filtration rate (eGFR), secondly to investigate two SNPs (+276 G>T (rs1501299) and +45 T>G (rs2241766)) in ADIPOQ gene, their possible association with CKD and their impact on plasma adiponectin levels.

## Methods

Seventy eight Egyptian pediatric patients with advanced CKD [stages 4 and 5 based on eGFR according to the National Kidney Foundation classification [National Kidney Foundation, 2002], [25] were included in the study. They were divided into two groups undergoing CT (defined as severe reduction in GFR ranged between 15 and 29 mL/min. per 1.73 m2) (n = 32) or MHD (defined as ESRD with GFR ranged between5-15 mL/min. per 1.73 m2) (n = 46). MHD children were selected from the hemodialysis unit of the Center of Pediatric Nephrology and Transplantation (CPNT), while CT children were selected from the nephrology pediatric clinic, Children’s Hospital, Cairo University. The study was done from June 2010 to December 2010. The inclusion criteria for MHD patients included patients with onset of hemodialysis (HD) below 16 years with at least 6 months duration on MHD. They were treated with hemodialysis for 3–4 h three times weekly with a polysulfone membrane using bicarbonate-buffered dialysate. The Duration of hemodialysis was 2.75±1.59 years.

Thirty two MHD patients and 16 CT patients were taking antihypertensive treatment. None of the patients was treated with fish oil. All patients were selected to be ambulatory, non obese, with BMI below 30 kg/m2 to avoid the potential confounding associations of obesity and cachexia with altered adipocytokine levels [26]. None of CKD patient in any group had cardiovascular events on the basis of examination and detailed clinical history. Additional exclusion criteria were liver disease and nephrotic syndrome (defined as daily proteinuria >3.5 g/1.73 m2).

All control subjects (n = 70) were healthy with no clinical signs of vascular or renal disease and no family history of renal disease as well as lack of medications taken at the time of the study. Control subjects were selected to be matched for age and gender to the patient groups, as well as within the same BMI limits. They were collected from the pediatric clinic of National Research Centre (NRC), Cairo, Egypt. An informed consent for genetic studies was obtained from parents of all participants. The protocol of the study was read and approved by the Ethics Committee of NRC in Egypt.

### Echocardiography

All echocardiographic measurements were performed according to the recommendations of the American Society of Echocardiography [27]. Left ventricular mass (LVM) was calculated according to the formula described by Devereux *et al.*[28] and was indexed to height^2.7^ (LVM index) [28]. The height-based indexing of LVM was specifically chosen to minimize potential distortions attributable to extracellular volume expansion (with surface area indexing being weight-sensitive). Left ventricular hypertrophy (LVH) was defined as LVMI greater than 51g/m^2.7^

### Biochemical markers

Venous blood samples were collected in the morning after an overnight fast on a midweek dialysis day, before the dialysis session. Three ml of venous blood sample was collected in EDTA vials for the extraction of genomic DNA. Complete blood count and pre- and post-dialysis kidney function test were determined by standard laboratory methods. Estimations of the plasma concentration of total cholesterol (TC), triglyceride (TG) and HDL cholesterol were made by using an Olympus AU400 (Olympus America, Inc., Center Valley, Pa., USA).

The determination of high sensitivity C-reactive protein (hs-CRP) in serum was performed by solid-phase chemiluminescent immunometric assay (Immulite/Immulite 1,000; Siemens Medical Solution Diagnostics, Eschborn, Germany) [29].

The assay of human IL6 in serum was performed by ELISA (Enzyme–Linked Immunosorbent Assay) method for quantitative measurement of serum IL6 (Ray Biotech, Inc.) [30].

Assay of serum adiponectin level was done using ELISA kit (Ray Biotech, Inc.) [31].

### Genotyping of ADIPOQ gene

**Figure 1:**
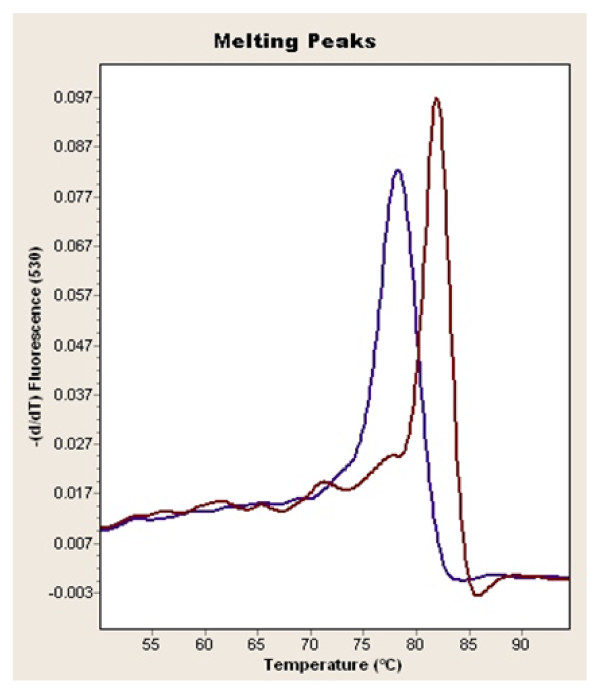
The homo mutant and the wild types of ADIPOQ 276 gene.

The analysis was carried out on a Light Cycler apparatus (Roche Molecular Biochemical). Primers of 45T>G SNP were 5′TCTCTCCATGGCTGACAGTG3′ and reverse primer 5′CCTTTCTCACCCTTCTCACC3′, while primers of 276G>T SNP were 5′GGCCTCTTTCATCACAGACC3′ and reverse primer 5′AGATGCAGCAAAGCCAAAGT3′ For SNP +45T>G and SNP +276G>T, fragments were amplified by rapid-cycling in a reaction volume of 20μl with 0.5μmol/l each primer, 0.15μmol/L anchor and syber green , 200 ng of genomic DNA, 1 ×DNA Master mix (Roche Molecular Biochemical) containing nucleotides, Taq DNA polymerase and 10 m m Mg^2+^. The final Mg^2+^ concentration in the reaction mixture was adjusted to 4 m m. The samples were loaded into glass capillary cuvettes (Roche Molecular Biochemicals) and centrifuged. After an initial denaturation step at 95°C for 5 min, amplification was performed using 50 cycles of denaturation (95°C for 3 s), annealing (57°C for 12 s for SNP +45T>G and 55°C for 12 s for SNP +276G>T) and extension (72°C for 10 s for SNP +45T>G and 72°C for 8 s for SNP +276G>T) on the Light Cycler. The temperature-transition rates were programmed at 20°C/s from denaturation to annealing, 20°C/s from annealing to extension and 20°C/s from extension to denaturation. Fluorescence was measured at the end of the annealing period of each cycle to monitor amplification. After amplification was completed, a final melting curve was recorded by heating to 95°C for 30 s with 20°C/s, holding at 40°C for 30 s with 20°C/s, and then heating slowly at 0.3°C/s until 80°C. Fluorescence was measured continuously during the low-temperature ramp. The fluorescence signal (F) was plotted in real time against temperature (T) to produce melting curves for each sample (F vs T). Melting curves were then converted to derivative melting curves by plotting the negative derivative of the fluorescence with respect to temperature against temperature (−(dF/dT) vs T). The entire process took approximately 30 min, with no separate manipulation of the product necessary [Figure 1[32].

### Statistical analysis

Statistical package for social science (SPSS) program version 16.0 was used for analysis of data. Data were summarized as mean±SD, range or percentage. The Kolmogorov-Smirnov test was used for evaluating the normality of data. For those data with abnormal distribution, log transformation was performed before a t-test. Matching was done by using two main approaches: pair (individual) matching and frequency matching. The number of subjects required for each subgroup was determined by Confidence Intervals. Power analysis was used to calculate the minimum sample size required to accept the outcome of a statistical test with a particular level of confidence. A sample size of 20 will give us approximately 80% power (alpha = 0.05, two-tail) to reject the null hypothesis of zero correlation. We used power calculations performed by the Power and Precision program (Biostat) to determine the number of chromosomes required to detect a significant difference between the polymorphism frequency in the reference population and the expected frequency. Power commonly sets at 80%; however, at that level, a polymorphism would be missed 20% of the time. Data were valuated between the experimental groups by One-Way Analysis of Variance (ANOVA) followed by Tukey’s multiple comparison tests. Allele and genotypic frequencies for ADIPOQ45 and 276 alleles were calculated with the gene counting method. Hardy–Weinberg equilibrium was tested with the *X*^*2*^ test. Comparison of the categorical data i.e. different ADIPOQ genotypes among patients was done by independent-samples *t* test where appropriate after evaluating the normality of data. Fisher’s exact test and *X*^*2*^ test were done to confirm the results. Multiple linear regression analysis was performed to assess the influence of metabolic risk factors and mutant alleles on adiponectin level. A p value of < 0.05 was considered significant.

## Results and Discussion

Anthropometric, clinical, and biochemical parameters in controls and CKD subjects were shown in Table 1. MHD, CT, and control subjects were comparable for gender, age and body mass index. The groups also had comparable blood pressure recordings. Patients receiving dialysis had higher absolute SBP and absolute DBP than healthy controls. Plasma creatinine concentration was higher by design in CKD groups with intermediate values in CT and highest levels in MHD. As expected, eGFR was lowest in MHD and within stages 4 and 5 for CT patients. It was observed that the lipid parameters namely TC, TG and HDL cholesterol, differ significantly among the CKD patients when compared to the controls (*p* < 0.05). Serum total cholesterol level was higher in MHD than in all other groups, while serum TG concentration was higher in CT than in both MHD and control subjects. Plasma hs-CRP was higher in MHD than in all other groups while serum IL6 and adiponectin levels were significantly higher in both stages of CKD children than in controls with increased level in CT children than in MHD group (*P* = 0.01). No significant difference between male and female CKD patients as regard to serum adiponectin level.

**Table 1:** Clinical and biochemical data of the studied groups

	CT	MHD	Controls
	(n = 32)	(n = 46)	(n = 70)
Age	9.14 ± 7.59	10.62 ± 3.49	10.7 ± 4.51
Gender (M/F)	15 (46.88%) /17(53.12%)	25(54.35%) /21(45.65%)	40(57.14%) /30(42.86%)
BMI(kg/m^2^)	17.64 ± 1.17	18.89 ± 3.00	20.88 ± 1.50
SBP (mmHg)	98.66 ± 6.66	127.13 ± 18.37 ^b^*	94.55 ± 9.80
DBP (mmHg)	64.66 ± 6.67	85.15 ± 13.76 ^b^*	60.59 ± 10.11
Creatinine (mg/dl)	3.93 ± 3.75 ^a*^	6.32 ± 1.55 ^b^*	0.77 ± 0.34
Predialysis urea, (mg/dl)	51.12 ± 10.45 ^a*^	71.56 ± 20.61 ^b^*	7.80 ± 2.64
eGFR, ml_min_1 _1.73m2	15.41 ± 1.76 ^a**^	11.30 ± 3.35 ^b^**	87 ± 8.9
Dialysis, Yrs		2.75 ± 1.59	
Nephropathies:			
Hereditary nephropathies	0	17	
renal hypoplasia or dysplasia	14	2	
obstructive uropathies	8	6	
neurogenic bladder	4	2	
Glomerulopathy	0	2	
Unknown	4	17	
Metabolic	2	0	
Antihypertensive drugs:			
*α*-adrenoceptor antagonists	2	2	
*ß*-blockers	0	9	
ACE inhibitors	6	17	
Ca channel blockers	10	29	
Kt/V		1.69 ± 0.42	
Total cholesterol (mg/dl)	164.44 ± 50.10	193.04 ± 51.37 ^b^*	160.31 ± 18.74
Triglycerides (mg/dl)	160.78 ± 57.33 ^a**^	147.00 ± 66.98 ^b^**	65.31 ± 18.35
HDL cholesterol (mg/dl)	21.35 ± 1.17^a*^	27.34 ± 9.88 ^b^*	39.55 ± 7.94
hs-CRP, mg/dl	3.04 ± 3.24	3.62 ± 3.97 ^b^*	1.36 ± 0.67
IL6(pg/ml)	59.50 ± 46.48 ^a*^	19.10 ± 23.68 ^b^**	3.17 ± 1.56
Adiponectin (μg/ml)	154.00 ± 7.18 ^a**^	52.44 ± 19.1 ^b^**	24.80 ± 8.23

Data was evaluated by ANOVA test. Values are presented as means ± SD or percentage as applicable. *CT* conservative treatment, *MHD* maintenance hemodialysis, *BMI* body mass index, *SBP* systolic blood pressure, *DBP* diastolic blood pressure, *eGFR* estimated glomerular filtration rate, *ACE* angiotensin converting enzyme, *Kt/V* adequacy of hemodialysis, *hs-CRP* high sensitivity c-reactive protein. ^a^*P < 0.05 or ^a^**P < 0.01vs. control and MHD ^b^*P < 0.05 or ^b^**P < 0.01 vs. control and CT .

Distributions of ADIPOQ +45 T>G and ADIPOQ +276G>T genotypes.

The genotype frequencies in each group satisfied the Hardy–Weinberg equilibrium. The distribution of genotype.

The frequencies of the TT, TG, and GG genotypes of +45 T>G gene were 100%, 0%, and 0% in both stages of CKD children and 100%, 0%, and 0% in the controls, respectively. The frequencies of the T and G alleles of +45 T>G gene were 100% and 0% in patients and 100% and 0% in the controls, respectively. This means that the wild type of ADIPOQ +45T>G gene (T) is the main allele for patients and controls and no significant difference was observed between them. MHD and CT patients had significantly higher frequency of the TT genotype of +276G>T gene (*P* = 0.04) compared with control subjects. The frequency of the T allele carriers was significantly higher in MHD than in healthy subjects. There was no significant difference between MHD and CT groups as regard to GT and TT genotypes (*P*>0.05) (Table 2).

**Table 2:** Frequencies of ADIPOQ genotypes in patients and controls

Gene		CT	MHD	Controls
		(n = 32)	(n = 46)	(n = 70)
+276 G>T Alleles	G	54(84.38%)	73(79.35%)	125(89.29%)
	T	10(15.62%)	19(20.65%) ^b^*	15(10.71%)
Genotypes	GG	24(75.00%)	32(69.56%)	55(78.57%)
	GT	6(18.75%)	9(19.57%)	15(21.43%)
	TT	2(6.25%) ^a^*	5(10.87%) ^b^*	0(0%)
+45 T>G Alleles	T	64(100%)	92(100%)	140(100%)
	G	0(0%)	0(0%)	0(0%)
Genotypes	TT	32(100%)	46(100%)	70(100%)
	TG	0(0%)	0(0%)	0(0%)
	GG	0(0%)	0(0%)	0(0%)

Data was evaluated by the gene counting method. Values were presented as percentage. *CT* conservative treatment, *MHD* maintenance hemodialysis. ^a^*P* < 0.05 vs. control and CT^, b^*P* < 0.05 vs. control and MHD.

In order to assess the cumulative effect of ADIPOQ+276G>T gene polymorphism with other risk factors; we compared various clinical parameters of the CKD patients between two genotypic groups, GG and GT+TT (Table 3). It was observed that neither mean age nor any of the lipid parameters differs significantly among the two sub-groups (*p* > 0.05). Serum adiponectin was significantly higher (*p* = 0.04) in the GT+TT group than in the GG group.

**Table 3:** Clinical and laboratory characteristics of CKD patients with different ADIOPQ 276 genotypes

	GG	GT+TT	P-value
	N = 56	N = 22	
Age Years	10.73 ±3.98	10.05 ± 3.15	0.51
LVMI	53.86 ± 9.67	50.86 ± 10.97	0.39
Cholesterol(mg/dl)	186.58 ± 47.22	178.20 ± 60.10	0.66
TG(mg/dl)	154.08 ± 71.73	134.71 ± 42.78	0.40
HDL cholesterol(mg/dl)	24.93 ± 11.29	28.15 ± 13.97	0.45
Adiponectin(μg/ml)	70.21 ± 42.85	90.92 ± 43.15	0.04*
hs-CRP(mg/dl)	3.54 ± 3.72	3.54 ± 4.07	0.99
IL6(pg/ml)	18.80 ± 25.23	24.87 ± 31.67	0.56

Significance was estimated using independent t-test. Data are means ± SD. *LVMI* left ventricular mass index, *TG* triglyceride, *hs-CRP* high sensitivity C - reactive protein, *IL6* interleukin 6. P is significant if < 0.05.

Correlation between serum adiponectin levels and different variables.

In the whole study population, a significant positive correlations was observed between plasma adiponectin and IL6 level (r = 0.43, *p* = 0.006), whereas a negative correlation was found between adiponectin level and hsCRP concentration (r = −0.27, *p* = 0.04). Plasma adiponectin correlated with levels of cholesterol (r = −0.47; *p* = 0.009) and HDL cholesterol (r = −0.61; *p* = 0.0001) levels, whereas no significant correlation (r = 0.18, *p* = 0.41) was observed between plasma adiponectin and serum triglyceride. No significant correlations were observed between serum adiponectin level, age, BMI or LVMI. Significant correlations were observed between serum adiponectin , eGFR and blood urea (r = 0.45, *p* = 0.04 and r = −0.28, *p* = 0.04) (Table 4).

**Table 4:** Pearson’s Correlation between adiponectin and different variables

	R	P value
Age	0.05	0.71
BMI	0.20	0.49
LVMI	−0.15	0.39
eGFR	0.45	0.04*
Urea	−0.28	0.04*
Cholersterol	−0.47	0.009**
TG	0.18	0.40
HDL-Cholesterol	−0.61	0.0001**
hs-CRP	−0.27	0.04*
IL6	0.43	0.006**
Mutant ADIPOQ276 gene	0.19	0.43

*BMI* body mass index, *eGFR* estimated glomerular filtration rate, *hs-CRP* high sensitivity c-reactive protein, *IL6* interleukin 6. *P < 0.05 or S**P < 0.001 were considered significant.

IL6 was positively correlated with blood urea nitrogen(r = 0.45, *p* = 0.007) and hs-CRP(r = 0.45, *p* = 0.005)

In a backward multiple linear regression model, including total cholesterol, HDL cholesterol, hs-CRP, IL6 and mutant ADIPOQ 276 gene, only IL6(β = 0.85, *p* = 0.001) was significantly associated with adiponectin serum levels (Table 5).

**Table 5:** Multiple linear regression analysis correlating adiponectin level to different variables

Dependent variables	ß	P-value
Cholesterol	−0.30	0.91
HDL cholesterol	0.09	0.41
hs-CRP	−0.18	0.27
IL6	0.85	0.001**
Mutant ADIPOQ 276 gene	0.09	0.74

*hs-CRP* high sensitivity c-reactive protein, *IL6* interleukin 6. **P < 0.01 was considered significant.

The present study was in agreement with earlier reports that plasma ADPN was elevated among patients with CKD compared to the general population and was related to several risk factors and inflammatory markers in a way consistent with the hypothesis that this protein might act as a protective factor for the cardiovascular system. On the other hand, genetic variations in the ADIPOQ gene seemed to have some impact on circulating adiponectin levels in these patients groups. The adiponectin level was higher in CT patients than in MHD patients. This might clarify that MHD patients were more exposed to cardiovascular risk factors. Previous reports notably agreed with the current data in indicating lack of increments of circulating adiponectin in renal patients undergoing hemodialysis compared with conservative treatment, in spite of presumable extreme reductions of renal function in the hemodialysis groups [33,34]. In the above studies, it is well possible that lack of plasma adiponectin increments in hemodialysis resulted from a decline in adipose tissue contribution with a parallel relative increment of passive accumulation [33,34].

Whereas hypoadiponectinaemia had been demonstrated for several groups of patients with increased risk for CVD [11,13,20-22], the present study demonstrated markedly elevated plasma adiponectin levels in CKD patients. Thus, our results confirmed those reported by Zoccali et al [7], who found markedly elevated adiponectin levels in a group of ESRD patients treated by HD. The reason(s) why ESRD patients had elevated levels of plasma adiponectin are not completely evident.

Chronic renal failure is one of the diseases state known to be associated with increased plasma ADPN concentrations; therefore, it represents a useful model for elucidation of the potential clinical implications of this substance. Physiologic concentrations of this substance exhibit inhibitory effects on TNF-α-induced monocyte adhesion and adhesion molecule expression [2]. There is evidence that atherosclerosis is strongly associated with inflammation among uremic patients [35,36]. If the inflammatory response detected in atherosclerotic lesions is effectively counteracted by ADPN *in vivo*, then this protein may have the potential for prevention and/or retardation of atherogenesis in various diseases, including chronic renal failure. The directions of the relationships between ADPN and several metabolic risk factors [36], such as cholesterol, HDL cholesterol and markers of inflammation (hs-CRP and IL6), were all in agreement with the hypothesis that ADPN may have a protective role for the cardiovascular system among CKD patients.

The present study provided an evidence of association between a variant in the adiponectin gene and CKD in children. MHD and CT patients had significantly higher frequency of the TT genotype of +276G>T gene compared with control subjects, while no significant difference was found in the distribution of ADIPOQ +45T>G gene polymorphisms between CKD patients and controls, suggesting that ADIPOQ276 gene polymorphisms may be a significant contributor to CKD susceptibility.

How does the 276G4T SNP affect the adiponectin gene function is still an open question. Although a possible effect on gene expression of SNPs with no apparently biological significance cannot be ruled out and, in fact, has been recently reported also for the adiponectin gene [35,36]. It appears more likely that this SNP is in linkage disequilibrium with another mutation either within, or in other genes close to, the adiponectin gene that determines its negative effects [32,37,38].

Our findings suggested that genetic variations of the adiponectin gene have a positive impact on plasma adiponectin levels in CKD patients. Our patients with the relatively rare GT+TT ADIPOQ 276 genotypes had significantly higher plasma adiponectin levels than the other genotypes, no significant differences in plasma adiponectin levels were noted for any of the other polymorphisms in this patient group. This finding was in agreement with data by Menzaghi et al [20] from non diabetic individuals showing significantly lower plasma levels of adiponectin in ADIPOQ 276 G/G homozygotes. Polymorphisms in the ADIPOQ gene had been shown to correlate with adiponectin serum levels in previous study in which the expression of the G allele at SNP +45 T>G was consistently higher than the T allele among all study subjects [39]. Moreover, Hara et al. reported that the G allele at SNP +276 G>T is inversely associated with lower plasma adiponectin concentration in Japanese population [18].

In the present study, a significant negative correlation was found between adiponectin and one of biomarkers of inflammation, hs-CRP and a positive correlation was observed between plasma IL-6 and ADPN after adjusting for CRP. While this observation might be explained by the co linearity between CRP and IL-6, it is pertinent that both ADPN and IL-6 are adipocyte derived products and their concordance could represent a co-secretory response after accounting for the variability in IL-6 levels that is represented by CRP. Importantly, the inverse correlation of adiponectin and hs-CRP remained statistically significant even after adjustment for confounders. Thus, our data suggested that hypoadiponectinaemia might also serve as a marker of increased inflammatory status in CKD patients. Our results confirmed those recently presented by Ouchi et al [12] who reported that patients with coronary atherosclerosis, showing a reciprocal association of adiponectin and CRP levels in human plasma. It is believed that adiponectin has anti-inflammatory properties and can counteract the pro-inflammatory effects of TNF-a [2] which might influence the production of IL-6 and CRP. A beneficial anti-inflammatory role of adiponectin is further supported by in vivo studies showing that adiponectin prevented neointimal formation [1].

## Conclusions

The present study revealed markedly elevated levels of plasma adiponectin in CKD pediatric patients. An association between the 276G>T SNP in the adiponectin gene and CKD in children was found. Genetic variations of +276G>T SNP in the adiponectin gene seemed to have a positive impact on plasma adiponectin levels. The complicated interaction between environmental factors and ADIPOQ gene polymorphisms in terms of susceptibility to CKD, especially in ethnically diverse populations has to be explored.

## Competing interests

The authors declare that they have no competing interests.

## Authors’ contributions

MFE and SMS carried out all samples collection and patients work up. MFE has interpretated the data, performed the statistical analysis and has written the manuscript.MMS was involved in the patients work up. EAE, NE, MRK, DK and GSE have performed the immunoassay and the gene polymorphism determination. All authors read and approved the final manuscript.
